# The effect of COVID rehabilitation for ongoing symptoms Post HOSPitalisation with COVID-19 (PHOSP-R): protocol for a randomised parallel group controlled trial on behalf of the PHOSP consortium

**DOI:** 10.1186/s13063-023-07093-7

**Published:** 2023-01-26

**Authors:** Enya Daynes, Molly Baldwin, Neil J. Greening, Thomas Yates, Nicolette C. Bishop, George Mills, Matthew Roberts, Malik Hamrouni, Tatiana Plekhanova, Ioannis Vogiatzis, Carlos Echevarria, Rashmita Nathu, Hamish J. C. McAuley, Lorna Latimer, Jennifer Glennie, Francesca Chambers, Ruth Penfold, Emily Hume, Dimitrios Magaritis, Charikleia Alexiou, Sebastian Potthoff, Mitchell James Hogg, Catherine Haighton, Bethany Nichol, Olivia C. Leavy, Matthew Richardson, Omer Elneima, Amisha Singapuri, Marco Sereno, Ruth M. Saunders, Victoria C. Harris, Claire M. Nolan, Charlotte Bolton, Linzy Houchen-Wolloff, Ewen M. Harrison, Nazir Lone, Jennifer Quint, James D. Chalmers, Ling-Pei Ho, Alex Horsley, Michael Marks, Krisnah Poinasamy, Betty Ramen, Louise V. Wain, Christopher Brightling, William D.-C. Man, Rachael Evans, Sally J. Singh

**Affiliations:** 1 NIHR Leicester Biomedical Research Centre-Respiratory, Leicester, UK; 2 Department of Respiratory Sciences, University of Leicester, Leicester, UK; 3 NIHR Leicester Biomedical Research Centre- Diabetes, Leicester, UK; 4 Diabetes Research Centre, College of Life Sciences, University of Leicester, Leicester, UK; 5 National Centre for Sport and Exercise Medicine, School of Sport, Exercise and Health Sciences, Loughborough University, Loughborough, UK; 6 Faculty of Health and Life Sciences, Northumbria University Newcastle, Newcastle upon Tyne, UK; 7 The Newcastle upon Tyne Hospitals NHS Foundation Trust, Newcastle, UK; 8 Department of Social Work, Education, and Community Wellbeing, Northumbria University Newcastle, Newcastle upon Tyne, UK; 9 Department of Health Sciences, University of Leicester, Leicester, UK; 10 College of Health, Medicine and Life Sciences, Brunel University, London, UK; 11 Harefield Respiratory Research Group, Guy’s and St Thomas’ NHS Foundation Trust, London, UK; 12 School of Medicine, The University of Nottingham, Nottingham, UK; 13 Centre for Medical Informatics, The Usher Institute, University of Edinburgh, Edinburgh, UK; 14 Usher Institute, University of Edinburgh, Edinburgh, UK; 15 National Heart and Lung Institute, Imperial College London, London, UK; 16 Royal Infirmary of Edinburgh, NHS Lothian, Edinburgh, UK; 17 MRC Human Immunology Unit, University of Oxford, Oxford, UK; 18 Division of Infection, Immunity & Respiratory Medicine, Faculty of Biology, Medicine and Health, University of Manchester, Manchester, UK; 19 Hospital for Tropical Diseases, University College London Hospitals, London, UK; 20 Division of Infection & Immunity, University College London, London, UK; 21 Asthma UK and British Lung Foundation, London, UK; 22 Radcliffe Department of Medicine, University of Oxford, Oxford, UK; 23 University of Dundee, Ninewells Hospital and Medical School, Dundee, UK; 24 Harefield Respiratory Research Group, Heart, Lung and Critical Care Clinical Group, Guy’s and St Thomas’ NHS Foundation Trust, London, UK

**Keywords:** COVID-19, Rehabilitation, Digital, Randomised controlled trial

## Abstract

**Introduction:**

Many adults hospitalised with COVID-19 have persistent symptoms such as fatigue, breathlessness and brain fog that limit day-to-day activities. These symptoms can last over 2 years. Whilst there is limited controlled studies on interventions that can support those with ongoing symptoms, there has been some promise in rehabilitation interventions in improving function and symptoms either using face-to-face or digital methods, but evidence remains limited and these studies often lack a control group.

**Methods and analysis:**

This is a nested single-blind, parallel group, randomised control trial with embedded qualitative evaluation comparing rehabilitation (face-to-face or digital) to usual care and conducted within the PHOSP-COVID study. The aim of this study is to determine the effectiveness of rehabilitation interventions on exercise capacity, quality of life and symptoms such as breathlessness and fatigue. The primary outcome is the Incremental Shuttle Walking Test following the eight week intervention phase. Secondary outcomes include measures of function, strength and subjective assessment of symptoms. Blood inflammatory markers and muscle biopsies are an exploratory outcome. The interventions last eight weeks and combine symptom-titrated exercise therapy, symptom management and education delivered either in a face-to-face setting or through a digital platform (www.yourcovidrecovery.nhs.uk). The proposed sample size is 159 participants, and data will be intention-to-treat analyses comparing rehabilitation (face-to-face or digital) to usual care.

**Ethics and dissemination:**

Ethical approval was gained as part of the PHOSP-COVID study by Yorkshire and the Humber Leeds West Research NHS Ethics Committee, and the study was prospectively registered on the ISRCTN trial registry (ISRCTN13293865). Results will be disseminated to stakeholders, including patients and members of the public, and published in appropriate journals.

**Article summary:**

Strengths and limitations of this study

• This protocol utilises two interventions to support those with ongoing symptoms of COVID-19

• This is a two-centre parallel-group randomised controlled trial

• The protocol has been supported by patient and public involvement groups who identified treatments of symptoms and activity limitation as a top priority

**Supplementary Information:**

The online version contains supplementary material available at 10.1186/s13063-023-07093-7.

## Administrative information

**Table:** 

Title	The effect of COVID rehabilitation for ongoing symptoms Post HOSPitalisation with COVID-19 (PHOSP-R): Protocol for a randomised parallel group controlled trial on behalf of the PHOSP consortium
Trial Registration	ISRCTN trial registry ISRCTN13293865 & ISRCTN10980107
Protocol version	1.1, 23 September 2021
Funding	Funding Jointly funded by UK Research and Innovation and National Institute of Health Research (grant references: MR/V027859/1 and COV0319) and by core funding provided by NIHR Leicester Biomedical Research Centre - a partnership between the University Hospitals of Leicester NHS Trust, the University of Leicester and Loughborough University and by pump priming funding provided by Northumbria University Newcastle (MDRT IHSC and HLS Faculty). Professor Singh is a National Institute for Health Research (NIHR) Senior Investigator. The views expressed in this article are those of the author(s) and not necessarily those of the NIHR, or the Department of Health and Social Care.
Author details	As listed on page 1-2
Name and contact for the trial sponsor	University of Leicester rgosponsor@leicester.ac.uk
Role of sponsor	Legal responsibility and governance of the PHOSP-COVID trial

## Introduction

### Background and rationale

The current coronavirus (COVID-19) pandemic, caused by infection with the severe acute respiratory syndrome coronavirus 2 virus (SARS-CoV-2) virus, has resulted in hundreds of thousands of people being admitted to hospital for acute medical management in the UK [1]. In 2020, approximately 15% of individuals diagnosed with COVID-19 required clinical support in a hospital setting, with severe cases requiring a prolonged stay in intensive care [2]. Importantly, recent data has demonstrated that over 70% of individuals are not fully recovered at 5 months and 1 year, and those discharged from hospital care are likely to have at least one persistent symptom, such as dyspnoea, fatigue, chronic cough, functional impairment or mental health impairments 4–6 months post-discharge which can last 2 years [3–5]. Individuals experiencing COVID-19 symptoms more than 12 weeks after initial infection are considered to have ‘post-COVID syndrome’ or ‘long-COVID’ [6]. Ongoing symptoms can lead to functional impairment, reduced exercise capacity and difficulty performing activities of daily living including return to work [3].

The exact mechanisms for post-COVID-syndrome and exercise intolerance are not entirely understood and could be a combination of a number of factors which are dysfunctional breathing pattern, fibrosis/structural lung changes resulting in breathlessness and activity limitation and deconditioning and symptom limitation. COVID-19 can result in a dysregulated and exhausted immune system [7]. Elevated concentrations of immune-derived markers of systemic inflammation, such as tumour necrosis factor-α (TNF-α), interleukin-6 (IL-6) and interleukin-8 (IL-8), have been observed up to 40 days after COVID-19. The severity of ongoing health impairments are associated with increased levels of inflammatory proteins including IL-6 5 months after hospital discharge [3].

Exercise and self-management rehabilitation programmes improve symptoms, such as breathlessness and fatigue, reduce the risk of hospitalisation, and increase health-related quality of life in other chronic conditions [8–10]. The benefits of pulmonary and cardiac rehabilitation provide protection from morbidity and mortality, whilst reducing clinical burden [11, 12]. Exercise training, in the context of rehabilitation programmes, represents a pragmatic therapy capable of improving the function of a range of biological systems, including the pulmonary, cardiovascular, neuromuscular, and immune system, by increasing exercise tolerance and reducing symptom burden [13].

Given the similarity between symptoms of individuals with post-COVID-syndrome and those with chronic diseases, it is plausible to propose that rehabilitation may convey a range of benefits following hospitalisation [6]. Early evidence supports this hypothesis, with 6 weeks of COVID rehabilitation resulting in an increase in exercise tolerance and improved respiratory symptoms, fatigue, and cognition in individuals with post-COVID-syndrome [14]. Five studies have been identified as delivering a rehabilitation intervention for COVID-19 in a systematic review with evolving evidence that rehabilitation can improve breathlessness and other post-COVID symptoms [15]. Importantly, the interventions focused on breathing exercises and low level physical activity advice, with little details on exercise prescription [15]. Attending face-to-face programmes can be challenging for some individuals particularly around work and with travel issues and therefore a suitable alternative such as digital interventions are required.

There is evidence to support an unsupervised telerehabilitation programme of aerobic exercises, breathing exercises and lower limb strengthening exercises following hospitalisation with COVID-19 [16]. This demonstrated improvements in exercise capacity measured by the 6-min walk test in those with moderate breathlessness (modified MRC 2-3) [16]. NHS-England has co-developed a website (www.yourcovidrecovery.nhs.uk) alongside people with lived experiences of COVID-19. An increasing body of evidence supports the use of digitally delivered rehabilitation programmes in long-term condition conditions that overcome several access barriers to traditional face-to-face programmes [17, 18]. Early data has shown that the use of the Your COVID Recovery programme can improve health-related quality of life and COVID-related symptoms in an uncontrolled sample [19].

This protocol describes a randomised controlled trial with embedded qualitative evaluation to investigate whether an 8-week rehabilitation programme is more effective at improving recovery, compared to usual care following hospitalisation with COVID-19. The protocol has been written in accordance with the Standard Protocol Items: Recommendations for Interventional Trials (SPIRIT) guidance [20] and was developed in response to the priority setting partnership alongside experts in rehabilitation and those with lived experience of COVID-19 [21].

### Objectives

#### Primary objective

The primary objective of this study is to determine whether rehabilitation (either face-to-face or digital) added to usual care increases physical function compared to usual care alone in individuals with ongoing symptoms following COVID-19 hospitalisation.

#### Secondary objectives

- To compare the efficacy of face-to-face and/or digital COVID-19 rehabilitation strategies to a usual care in improving exercise capacity and symptoms in individuals following a COVID-19 hospitalisation
- Investigate the effect of face-to-face rehabilitation vs. usual care on immune cell counts, inflammatory cell phenotypes and stimulated-immune cell inflammatory cytokine release in blood biomarkers
- To understand the skeletal muscle response to rehabilitation in patients with ongoing post-COVID symptoms, including metabolic, gene/protein expression, and inflammatory changes
- To understand barriers and facilitators to the delivery and implementation of COVID rehabilitation (digital and face-to-face) in people with ongoing post-COVID symptoms, and staff delivering the service

## Methods

### Trial design and setting

**Fig. 1:**
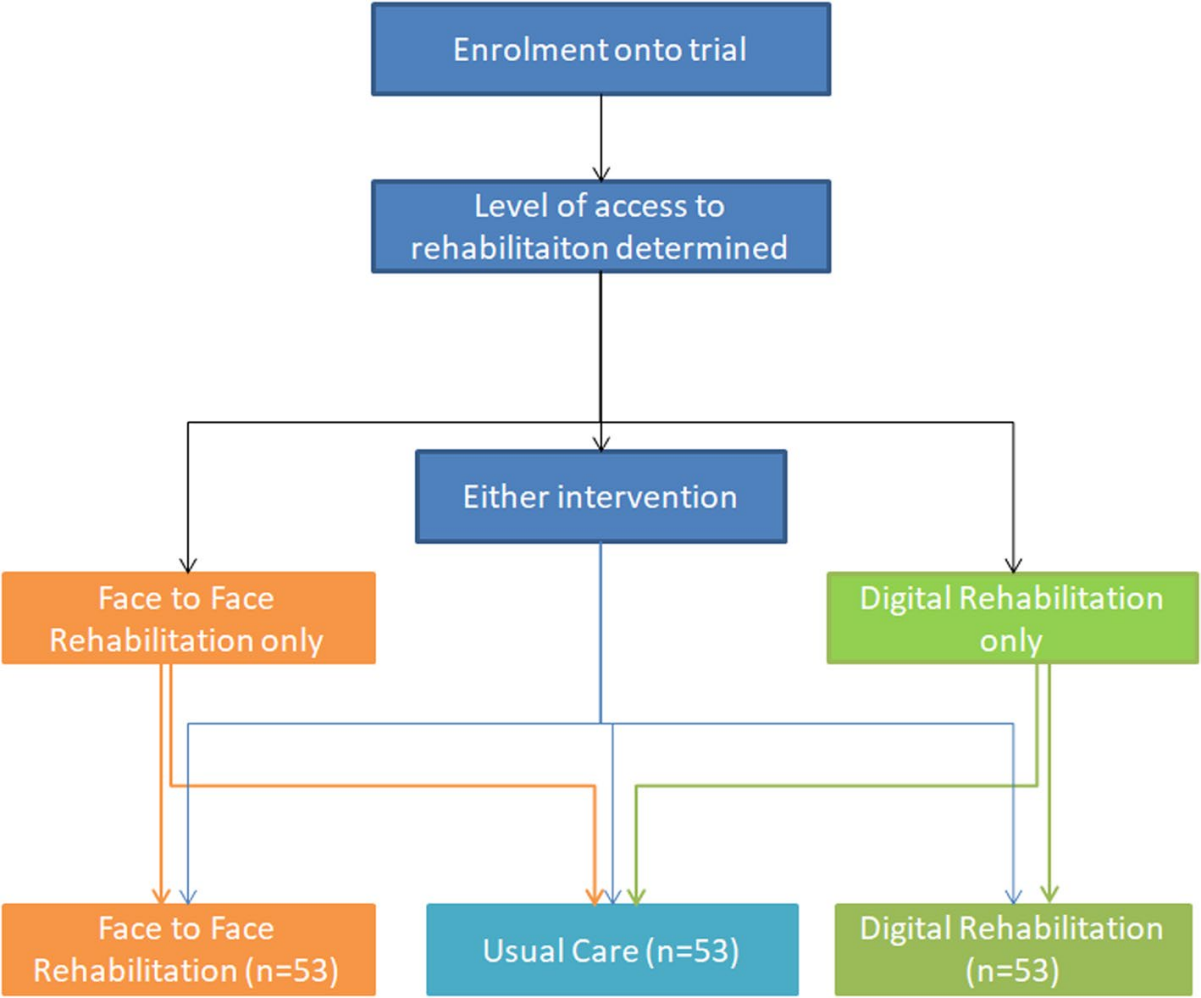
Randomisation process for PHOSP-R trial

This study is delivered in two UK centres and is single-blind parallel group randomised controlled trial. This trial aims to assess superiority of the interventions compared to a control (see Fig. 1). This study is a sub-study to the PHOSP-COVID study detailed elsewhere [3] (ISRCTN10980107).

### Participants—eligibility criteria

Participants are eligible if they were admitted to hospital during the acute phase of their COVID-19 confirmed (by PCR) or clinician diagnosed COVID-19 and have ongoing symptoms lasting more than 12 weeks, with no upper limit, that may be modifiable by a rehabilitation programme. These symptoms include reduced activity/exercise tolerance, fatigue, dyspnoea, musculoskeletal pain, short-term memory loss and a slowing down in thinking [14].

Individuals with a contraindication for exercise, as documented in the American College of Sports Medicine guidance, symptoms indicative of another medical condition that require further investigation/management or unstable comorbidities that require follow-up (i.e. palpitations), or who have completed COVID-19/exercise rehabilitation in the preceding 6 months will be excluded from the study. Those who were not admitted to hospital during their SARS-Cov-2 infection will be excluded [22]. Individuals must be willing to attend the face-to-face rehabilitation programme and/or access the digital rehabilitation programme to participate in this study. Those with a diagnosis of post exertional malaise or in the absence of a diagnosis but experiences severe debilitating fatigue (home or bed bound) that worsens with activity will not be recruited to this study.

### Who will take informed consent

Eligible participants will provide written informed consent alongside the delegated research study staff prior to being randomised into a study group.

### Additional consent provisions for collection and use of participant data and biological specimens

Participation in sub-studies is indicated on the consent form. If the participant agrees, the study team will request consent for review of the participants’ medical records and for the collection of blood samples to assess inflammatory markers and skeletal muscle biopsies. An additional clinical consent form is completed for muscle biopsies as it is an invasive procedure.

### Interventions

All groups receive usual care as defined by the National Institute for Clinical Excellence COVID-19 guidance.

### Face-to-face rehabilitation

This is an individualised symptom titrated programme of exercise, education and self-management. Individuals will complete up to five sessions per week, two of which are supervised sessions, for 8 weeks (approximately 90–120 min per session), delivered by healthcare professionals. The rehabilitation programme will consist of aerobic exercise (i.e. treadmill/ground walking at approximately 80% of incremental shuttle walk test (ISWT) speed where tolerated, cycling on a cycle ergometer) and resistance exercise training (upper and lower limb strength exercises). The exercise intensity and duration is tailored to the individuals current abilities, assessed in their first visit and calculated using a predicted VO_2_ max determined by the ISWT. In addition to the supervised sessions, patients will be asked to perform home-based exercise sessions which mimic the supervised sessions: three aerobic exercise sessions and one resistance exercise session per week which are recorded in a self-reported diary. This is used in conjunction with self-reported symptoms and will be used to determine exercise modifications and progression. Participants will be monitored for their symptoms during the programme and recorded in a self-reported diary, which includes the Borg and Rate of Perceived exertion. The programme will be modified in line with their symptoms and either increased or decrease the intensity based on their responses, including how they felt the following day(s). The exercises and respective symptom scores will be kept in a paper diary by the participant and monitored throughout the programme by a healthcare professional. Signs of post exertional malaise and post exertional symptom exacerbation will be monitored in discussion with the participant and healthcare professional at the start of each session and adjustments made as necessary. Participants will record their home sessions in the diary, and this will be reviewed at every session.

Each rehabilitation session will conclude with an educational discussion (approx. 30–60 min) delivered by a member of the multidisciplinary team. These discussions will be facilitated by information sheets from the Your COVID Recovery open access website (www.yourcovidrecovery.nhs.uk). Topics covered will include getting moving again, managing activities of daily living, breathlessness, fatigue management and recognising symptoms of worsening in response to exercise, fear and anxiety, mood and coping, memory and concentration, cough, eating well, sleeping hygiene, goal setting, headaches, managing symptom exacerbation and fluctuations, returning to work, question and answer session and next steps.

### Digital rehabilitation

Individuals will be given access to the password protected Your COVID Recovery® digital rehabilitation programme. This programme is structured to guide individuals through four stages; each stage houses specific tasks that individuals must complete to progress to the next stage and lasting approximately 2 weeks per stage. These tasks will include creating and updating goals, recording physical activity, recording symptoms, and viewing educational content. The programme contains interactive resources for managing COVID symptoms.

A healthcare professional will contact participants via telephone every 2 weeks (total of three calls) to discuss participant progress, review participant goals, COVID-19 symptoms (including symptom exacerbation and post exertional malaise) and current exercise prescription. In addition, individuals can contact a healthcare professional for support using the message function (monitored during working hours) or engage in peer-to-peer support via the Your COVID Recovery® site’s forum.

### Usual care (control)

Participants in the control arm will receive usual care for 8 weeks. Routine clinical care will continue such as medical follow-up, mental health services and other specialist services. After their involvement in the trial has concluded, individuals allocated to this group will receive their preferred rehabilitation strategy (i.e. face-to-face or digital rehabilitation).

### Criteria for discontinuing/modifying interventions

The intervention will be discontinued if the participant withdraws consent or if a change in circumstance results in the participant meeting the trial exclusion criteria and becomes unsafe to engage in the intervention; these participants will remain in the analysis for data collected up until the date of withdrawal. The intervention is individualised, and progression/modification will be determined through discussions with a patient and healthcare professional. Reasonable adaptations to exercise will be made based on clinical judgement and participant need. Implementing an 8-week rehabilitation programme or usual care will not require alteration to usual care pathways (including use of any medication), and these will continue for both trial arms.

### Provisions for post-trial care

There is no anticipated harm and compensation for trial participation. Those completing the trial will be offered their preferred form of rehabilitation and will continue with their usual care.

### Outcomes

All outcomes will be performed pre and post the intervention phase which is 8 weeks in total. Baseline demographics will be collected as well as information regarding their treatment during hospitalisation, social history (such as employment status, smoking, social deprivation) and past medical history.

#### Primary outcome measure

##### Incremental shuttle walk test

The primary outcome measure for this trial is the absolute change in the incremental shuttle walk test (ISWT) distance after the intervention phase. This is a measure of exercise tolerance [23]. To derive this variable, the ISWT will be performed in line with technical standards [24]. Briefly, this test is an externally paced, incremental test that requires patients to walk around a 10-m course at a speed dictated by audio. The walking speed progressively increases each minute, for a maximum of 12 min, with the test terminated when the patient is no longer able to keep up with the target walking speed. Individuals will perform the ISWT twice pre-intervention for familiarisation purposes with the highest distance achieved used for exercise prescription and once post-intervention.

#### Secondary outcome measures

Secondary outcome measures include physical measures (short physical performance battery, handgrip strength, maximum isometric quadriceps strength and physical activity) and questionnaires (EuroQol five-dimension five-level questionnaire, including the EuroQoL Visual Analogue Scale, Patient Health Questionnaire (PHQ9), the Generalised Anxiety Disorder (GAD7) 7-item scale, Dyspnoea-12, the modified MRC Dyspnoea scale used with the permission of the Medical Research Council, SARC-F, the Functional Assessment of Chronic Illness Therapy Fatigue Scale (FACIT), the Brief Pain Inventory, the General Practice Physical Activity Questionnaire (GPPAQ), the Nottingham Extended Activities of Daily Living questionnaire, the Montreal Cognitive Assessment (MOCA), the DePaul Symptom Questionnaire, and the Nijmegen Questionnaire) [24–34] (Table 1). Outcomes are further detailed in Supplement 2.

**Table 1:** Outcome measures

Outcome	Timepoint
Incremental Shuttle Walking Test (ISWT)	Primary
Short Physical Performance Battery (SPPB)	Secondary
Quadriceps Maximal Voluntary Contraction (QMVC)	Secondary
Handgrip strength	Secondary
Physical activity	Secondary
EuroQol 5 domain 5 level (EQ5D-5L)	Secondary
Patient Health Questionnaire (PHQ-9)	Secondary
Generalised Anxiety Disorder (GAD-7)	Secondary
Dyspnoea 12 (D12)	Secondary
SARC-F	Secondary
Modified Medical Research Council (MRC) dyspnoea scale	Secondary
Functional Assessment of Chronic Illness Therapy- Fatigue Scale (FACIT-FS)	Secondary
General Practice Physical Activity Questionnaire (GPPAQ)	Secondary
Nottingham Extended Activities of Daily Living (NEADL)	Secondary
Depauls Symptom Questionnaire (DSQ)	Secondary
Montreal Cognitive Assessment (MoCA)	Secondary
Nijmegen questionnaire	Secondary
Blood markers	Exploratory—optional
Muscle biopsies	Exploratory—optional

### Assessment of inflammatory markers

This is an optional outcome measure for patients. Before and after the intervention period, venous blood samples will be drawn into blood collection tubes containing EDTA and sodium heparin as anticoagulants. This will be in a sub-group of patients randomised to either the control or face-to-face group and will be an optional part of the trial.

### Skeletal muscle biopsies

This optional outcome measure for a subgroup of participants who volunteer to undergo this procedure will involve muscle biopsies of vastus lateralis taken pre- and post-intervention using the microbiopsy technique (Magnum, Bard). Approximately 100 μg of skeletal muscle will be taken and processed by snap freezing in liquid nitrogen for molecular analysis and embedded in optimal cutting temperature compound (OTC) (Sakura, USA) medium for histochemistry.

### Uptake, compliance and completion

Recruitment rate will be assessed to determine uptake and the reason for declining will be recorded. The number of face-to-face sessions will be recorded and analysed. For the digital intervention, the phases completed and the number of logins to the website will be recorded. Attendance to the phone calls will also be recorded. Reasons for withdrawal will be obtained where possible to do so and those who withdraw will not have completed follow-up data.

### Qualitative interviews

Semi-structured interviews will be conducted with participants and staff members to explore the benefits and challenges of COVID rehabilitation. This will be conducted by a researcher independent from the trial and the interventions. These will be performed on a 1:1 interview basis either virtually or face-to-face depending on preference. A purposive sample of patients who have completed the entirety of their intervention period will be recruited. Details of those participants who consent to be contacted will be passed to the qualitative interview research team who will approach participants via e-mail with the opportunity of taking part in either a face to face or virtual interview. All interviews will be audio recorded with permission and transcribed anonymously to ensure that all identifiable information is removed, managed using NVivo (version 12; QSR International). All analyses will be conducted according to the standard procedures of rigorous qualitative analysis through the use of reflexive thematic analysis [35, 36].

### Sample size

The sample size is calculated on the ISWT (primary outcome) with a change of 50 m at 90% power, with a standard deviation of 72 m and a 0.05 type 1 error as previously documented in the literature as the minimum important difference and variance of the ISWT [14, 37]. This requires 44 participants per group, 132 participants in total. The sample size has been inflated by 20% to account for attrition, and therefore, 159 participants will be recruited across two sites. This sample size also ensures the trial is fully powered for the FACIT and EQ5D questionnaire.

### Recruitment

Potential participants will be identified through the PHOSP-COVID study or by referral to COVID Rehabilitation Services from Long COVID assessment services. For the former, individuals who have consented to the PHOSP-COVID study and provided consent to be contacted about other research will be contacted by a member of the research team and sent a patient information sheet. For the latter pathway, participants will be recruited from COVID rehabilitation services waiting list at University Hospitals of Leicester NHS Trust and Newcastle upon Tyne Hospitals NHS Foundation Trust (both UK). All hospitalised patients are followed up through the Long COVID assessment service, irrespective of their involvement in PHOSP, and are screened by clinicians and researchers and deemed safe to undergo an exercise intervention. Individuals experiencing persistent COVID-19 symptoms will be referred to this pathway as part of their discharge follow-up and hospitalisation status will be screened by the researcher. Potential participants will be identified and approached by a member of their direct clinical care team and asked if a member of the research team can contact them to discuss the trial and give them a patient information sheet.

### Randomisation

Prior to randomisation, the level of access will be determined based on which interventions participants are able to do by asking participants whether they are able to attend face-to-face rehabilitation and/or access the digital rehabilitation programme. Individuals who can participate in either intervention will be randomised on a 1:1:1 ratio for face-to-face, digital or control (usual care). Participants who can only participate in one of the two interventions will be randomised 2:1 to the intervention or control (usual care) (Fig. 1). Randomisation will be generated within the online randomisation software ‘Sealed Envelope’ (https://www.sealedenvelope.com), using block randomisation with a stratified block of six participants. Researchers involved in delivering the rehabilitation programmes will implement the randomisation on a sealed envelope and assign participants.

### Blinding

Members of the research team assessing outcome measures will be blinded to the outcome of randomisation. There are no perceived circumstances where blinding will be necessary. Any unintentional blinding will be recorded and monitored.

### Data management

Data will be entered into the REDCap electronic data capture tool hosted at the University of Leicester before being transferred to a central data safe haven managed by the University of Edinburgh and eDRIS at Public Health Scotland. Users will be granted permission to use this data within the national safe-haven. Data will be quality checked and linked to their original PHOSP-COVID ID, if applicable, and data to obtain baseline characteristics and hospitalisation data. Data will be quality checked by a second researcher, and any outliers will be explored and removed as appropriate.

### Statistical analysis

#### Primary outcome

The statistical analysis will be performed in R studio and analysis will compare an intervention (face-to-face or digital) to the usual care group. Changes in the primary outcome (ISWT distance) will be compared across each arm using a *t*-test. Those with primary outcome data will be analysed in the group to which they were randomised. Two sensitivity analyses will be conducted for the primary outcome: (1) missing data will be imputed using multiple imputation allowing for a full ITT analysis and (2) a per-protocol analysis with those that adhere to the intervention defined if they attended 75% of face-to-face sessions or reach stage four of the digital intervention and attends the follow-up appointment.

Secondary outcomes will not be powered but will be performed in R studio and compare the interventions (face-to-face or digital) to the usual care group. This includes physical measures and questionnaires and will be performed using a *t*-test or non-parametric equivalent. Categorical data will be explored using a chi-squared test. Muscle biopsy data will be analysed in R Studio as above. The FDR adjustment for multiple comparisons with be applied to multiple comparisons [38]. Blood biomarkers will be analysed in R studio using linear mixed models, with intervention group (control vs face-to-face), time point (pre vs post) and group*time treated as fixed effects, and the participant identifier treated as a random effect.

Adverse events will be reported for each group. Uptake to the trial, and compliance to the interventions will be reported, and compared across groups (face-to-face vs. digital rehabilitation).

### Adverse event reporting

Any and all untoward events arising from the intervention that require further medical attention and/or hospitalisation will be recorded on an adverse events or serious adverse events log in the investigator site file and reported to the sponsor. Adverse events will be explored and categorised as related or unrelated to the trial intervention. Due to the nature of the trial, there are no formal stopping rules as problems that are detrimental to the participant are not anticipated.

### Frequency and plans for auditing conduct

The PHOSP consortium have regular study meetings in which this substudy would be discussed. The executive committee meet every 2 months, and this includes representation from funders and sponsors. Data meetings and PHOSP-Rehab steering meetings occur monthly. Additional meetings are arranged as required.

### Patient and public involvement

Patient and public involvement have been integral to the PHOSP-COVID study and consortium since conception. The PHOSP PPI group is co-chaired by NOCRI and BLF/Asthma UK with representation of over 10 relevant charities. Members of the ‘Long-COVID Facebook support group’ are closely involved and a Leicester BRC PPI group consisting of people with lived experience of a hospital admission for COVID-19. Patients and public are embedded within the PHOSP infrastructure including our working groups, core management group and executive and steering groups. Patients were involved in the development of the clinical research study including the overarching aims, choice of outcomes, consent processes and the structure of the study visits. Patients review all patient facing material. We have recently completed a joint patient and clinician research priority questions exercise hosted by advisors from the James Lind Alliance to ensure co-ownership of the direction of PHOSP-COVID research. The patient and public involvement groups will continue to support the implementation of this project as well as data interpretation and dissemination in the future and have assisted in the preparation of this manuscript. Additionally, this protocol was developed with key working groups in the PHOSP consortium including the Rehabilitation, Brain, Cardiac and Sarcopenia working group.

## Discussion

COVID-19 is a multi-system condition, characterised by a myriad of physical and mental health symptoms in the presence of reduced exercise tolerance. Together, these manifestations can compromise the ability to perform tasks of daily living, adversely impacting health-related quality of life [3]. This manuscript presents a detailed description of a multicentre, single-bind, parallel group, randomised controlled trial, designed to determine the effect of COVID-19 rehabilitation after a COVID-19 hospitalisation on disease symptoms, exercise tolerance, health-related quality of life and inflammation. The COVID-19 rehabilitation programme described in this protocol has been designed to be amenable to all real-world rehabilitation settings.

The outcomes of this trial will determine whether rehabilitation (face-to-face or digital) can improve exercise capacity in people with ongoing COVID-19 symptoms compared to usual care alone. Secondary outcomes will determine the impact on symptoms and health related quality of life. Exploratory outcomes aim to understand the effect of rehabilitation on immune biomarkers and skeletal muscle function and may provide further insight into mechanistic characteristics that contribute to ongoing symptoms and the potential response to exercise interventions, therefore providing a scientific hypothesis for the differences that may occur following rehabilitation. Qualitative interviews will allow for the in-depth understanding of the benefits of rehabilitation for participants and the potential barriers for staff delivering this intervention.

Adverse events will be carefully monitored with particular attention to those displaying signs of post exertional malaise (PEM) and post exertional symptom exacerbation (PESE). Steps have been taken to ensure those with known PEM/PESE are not recruited to this trial to ensure their symptoms are not worsened by rehabilitation. If it appears that someone is experiencing PEM/PESE during the intervention phase, then they will be withdrawn from the trial, upon discussion with the individual, and referred to the appropriate clinic(s). All adverse events will be documented and reported within the results.

This trial will be conducted across two UK centres and is reflective of those presenting to hospital with COVID-19. It is beyond the scope of this work to recruit those who did not present to hospital with COVID-19, though the authors acknowledge this is an important area of research. The results of this trial will allow for development of other rehabilitation services in the UK to support those with ongoing symptoms of COVID-19 and could contribute to the current small body of evidence for interventions for those with ongoing symptoms. There is a need to multiple, flexible interventions to suit the diverse needs of the population, for which there is small cohort data to support the potential benefit of rehabilitation. In the event of a negative trial, the results and interviews may help inform further intervention development and future research into this area.

### Trial progress

Trial recruitment commenced in March 2022 and is due to complete in March 2023. Current protocol version 1.1.

## Supplementary Information

**Additional file 1: Supplement 1.** Full PHOSP consortium.

**Additional file 2: Supplement 2.** Outcomes.

## Data Availability

The trial findings will be presented at conferences and published in peer-reviewed journals. Authorship will comply with the ICMJE recommendations. Direct access to the dataset will be granted to authorised representatives from regulatory authorities, authorised individuals from host organisations, funding bodies, the sponsor (University of Leicester) and from the University Hospitals of Leicester NHS Trust for monitoring and/or audit of the trial to ensure compliance with regulations. Requests for the use of data and samples can be made directly to the trial investigator and will be reviewed on a case-by-case basis by the trial team.
