# Supplementary material for: The effect of COVID rehabilitation for ongoing symptoms Post HOSPitalisation with COVID-19 (PHOSP-R): protocol for a randomised parallel group controlled trial on behalf of the PHOSP consortium
Source: Trials. 2023 Jan 26;24:61. doi: 10.1186/s13063-023-07093-7 (PMC9879232; doi:10.1186/s13063-023-07093-7)
Supplement: Supplementary file 2 — Additional file 2: Supplement 2. Outcomes. [file 13063_2023_7093_MOESM2_ESM.pdf]

Physical measures

Short physical performance battery

The short physical performance battery assesses lower extremity function (UCSF Division of Geriatrics 2000) and is a surrogate marker of frailty. The battery has three domains, balance, usual gait speed and lower limb strength which are measured by a three stage balance test (feet shoulder width apart, semi-tandem, and tandem position), 4m gait speed test, and a five repetition sit-to-stand test, respectively[1]. Participants can score a maximum of 12 points from the three assessments, with a higher score indicating a greater level of physical function.

Muscle strength assessments

Maximum handgrip strength will be measured using a dynamometer (JAMAR, UK), performed three times on both the dominant and non-dominant hand. The best score is taken per hand. Maximal isometric quadriceps strength will be measured using an isometric dynamometer on the dominant leg. The participant will have three practice attempts at 50%, 60% and 80% effort and three true attempts at 100% effort. The best attempt will be analysed.

Physical activity and sleep

Physical activity will be measured using wrist worn accelerometers worn on the non-dominant wrist (GENEActive Original, Activinsights Ltd, Kimbolton, UK) 24 hours per day, for a total of four weeks. This will be broken down into 2 x 14-day periods. The first period will be one week before and one week into the intervention. The second period will be during the final week of the intervention and the week after completing the intervention. A valid physical activity measurement is defined as a minimum of three days, with a valid day defined as at least 16 hours of wear[2]. Measures of physical activity such as average step count, total physical activity, intensity distribution, time inactive, in light-intensity and moderate-to-vigorous intensity, and the intensity of the most active 5, 10 and 30 minutes of the day will be extracted. In addition, total sleep time, the duration of the sleep window and sleep efficiency will be derived. Accelerometer data will be processed using the open-source R-package GGIR[3]. The data will then be cleaned as previously reported[4].

Questionnaires

The following questionnaires will be used to assess patient self-reported health-status pre- and post-intervention: EuroQol five-dimension five-level questionnaire, including the EuroQoL Visual Analogue Scale, Patient Health Questionnaire (PHQ9), the Generalised

Anxiety Disorder (GAD7) 7-item scale, Dyspnoea-12, the modified MRC Dyspnoea scale used with the permission of the Medical Research Council, SARC-F, the Functional Assessment of Chronic Illness Therapy Fatigue Scale (FACIT), the Brief Pain Inventory, the General Practice Physical Activity Questionnaire (GPPAQ), the Nottingham Extended Activities of Daily Living questionnaire, the Montreal Cognitive Assessment (MOCA), the DePaul Symptom Questionnaire, and the Nijmegen Questionnaire.

Assessment of inflammatory markers

This is an optional outcome measure for patients. Before and after the intervention period, venous blood samples will be drawn into blood collection tubes containing EDTA and sodium heparin as anticoagulants. This will be in a sub-group of patients randomised to either the control or face-to-face group, and will be an optional part of the trial. Further details are available in the online supplement. Heparinised whole blood will be used for flow cytometric determination of immune cell subsets and for isolation of peripheral blood mononuclear cells (PBMCs) for stimulation with lipopolysaccharide (LPS) and staphylococcal enterotoxin B (SEB). EDTA whole blood will be used for analysis of total and differential leukocyte counts with an automated haematology analyser.

Flow cytometry

Monocytes and lymphocytes will be gated based on forward vs. side scatter area and fluorescently conjugated antibodies will be used to identify classical, intermediate and non-classical monocyte subsets, T cells (including helper and cytotoxic subsets) and their naïve and memory subsets, and pro- and anti-senolytic NK cells (Table 2). Fluorescence minus one controls will be used to gate the abovementioned subsets. The proportions of the different subsets will be used with differential leukocyte counts to calculate the circulating numbers for each subset. Samples will be prepared using standard staining methods and data will be acquired using a 4-colour flow cytometer (Accuri C6, BD, Oxford, UK).

Peripheral Blood Monocyte Cell (PMBC) isolation and stimulation

Heparinised whole blood diluted in a 1:1 ratio with Dulbecco's phosphate buffered saline (D-PBS) will be layered onto Histopaque-1077, from which PBMCs will be derived using density gradient centrifugation. The number of viable PBMCs in the sample will be determined using a standard haemocytometer and trypan blue staining. On a sterile 24-well cell culture plate PBMCs in cell culture media will be seeded at 170,000 cells per well and treated with 100ng/ml of either LPS or SEB. After incubation for 24h at 37.0°C, 5% CO_2, the supernatants will be collected and frozen at -80°C for later determination of TNF-α, IL-6, and IL-10 concentrations using enzyme-linked immunosorbent assay (ELISA) kits.

Table 2. Antibody panels

Antibody	Fluorochrome	Supplier	Clone	Isotype
Monocytes				
CD14	FITC	BD Biosciences	M5E2	Mouse IgG2a, κ
CD16	PE	BD Biosciences	3G8	Mouse BALB/c x DBA/2
T cells				
CD3	FITC	BioLegend	UCHT1	Mouse IgG1, κ
CD27	PE	BD Biosciences	M-T27	Mouse BALB/c IgG1, κ
CD4	PE-Cy7	BD Biosciences	SK3	Mouse BALB/c IgG1, κ
CD8	PE-Cy7	BD Biosciences	RPA-T8	Mouse IgG1, κ
CD45RA	APC	BD Biosciences	HI100	Mouse IgG2b, κ
NK cells				
CD3	FITC	BioLegend	UCHT1	Mouse IgG1, κ
CD159a (NKG2A)	PE	BioLegend	S19004C	Mouse IgG1, κ
CD56	PE-Cy7	BD Biosciences	B159	Mouse IgG1, κ
CD314 (NKG2D)	APC	BioLegend	1D11	Mouse IgG1, κ

1. Guralnik et al., 2000 UCSF Division of Geriatrics. Short Physical Performance Battery (SPPB). Available from: http://www.youtube.com/watch?v=N_rJOGhQqZ4[last accessed 14/09/20]
2. Demeyer H, Mohan D, Burtin C, Vaes AW, Heasley M, Bowler RP, Casaburi R, Cooper CB, Corriol-Rohou S, Frei A, Hamilton A, Hopkinson NS, Karlsson N, Man WD, Moy ML, Pitta F, Polkey MI, Puhan M, Rennard SI, Rochester CL, Rossiter HB, Sciurba F, Singh S, Tal-Singer R, Vogiatzis I, Watz H, Lummel RV, Wyatt J, Merrill DD, Spruit MA, Garcia-Aymerich J, Troosters T. Chronic Lung Disease Biomarker and Clinical Outcome Assessment Qualification Consortium Task Force on Physical Activity. Objectively Measured Physical Activity in Patients with COPD: Recommendations from an International Task Force on Physical Activity..Chronic Obstr Pulm Dis. 2021 Oct 28;8(4):528-550. doi: 10.15326/jcopdf.2021.0213.
3. Migueles JH, Rowlands A V., Huber F, Sabia S, van Hees VT. GGIR: A Research Community–Driven Open Source R Package for Generating Physical Activity and

Sleep Outcomes From Multi-Day Raw Accelerometer Data. J Meas Phys Behav. 2019;2(3):188–96.

4. Plekhanova, T., Rowlands, A.V., Evans, R.A. et al. Device-assessed sleep and physical activity in individuals recovering from a hospital admission for COVID-19: a multicentre study. Int J Behav Nutr Phys Act 19, 94 (2022). https://doi.org/10.1186/s12966-022-01333-w
